# Interoperable IoMT Approach for Remote Diagnosis with Privacy-Preservation Perspective in Edge Systems

**DOI:** 10.3390/s23177474

**Published:** 2023-08-28

**Authors:** Erana Veerappa Dinesh Subramaniam, Kathiravan Srinivasan, Saeed Mian Qaisar, Paweł Pławiak

**Affiliations:** 1 Department of Computer Science and Business Systems, Ramco Institute of Technology, Rajapalayam 626117, India; dinesh@ritrjpm.ac.in; 2 School of Computer Science and Engineering, Vellore Institute of Technology, Vellore 632014, India; kathiravan.srinivasan@vit.ac.in; 3 CESI LINEACT, 69100 Lyon, France; smianqaisar@cesi.fr; 4 Electrical and Computer Engineering Department, Effat University, Jeddah 22332, Saudi Arabia; 5 Department of Computer Science, Faculty of Computer Science and Telecommunications, Cracow University of Technology, Warszawska 24, 31-155 Krakow, Poland; 6 Institute of Theoretical and Applied Informatics, Polish Academy of Sciences, Bałtycka 5, 44-100 Gliwice, Poland

**Keywords:** Internet of Medical Things (IoMT), patient privacy, security, authentication, clustering, encryption, routing

## Abstract

The emergence of the Internet of Medical Things (IoMT) has brought together developers from the Industrial Internet of Things (IIoT) and healthcare providers to enable remote patient diagnosis and treatment using mobile-device-collected data. However, the utilization of traditional AI systems raises concerns about patient privacy. To address this issue, we present a privacy-enhanced approach for illness diagnosis within the IoMT framework. Our proposed interoperable IoMT implementation focuses on optimizing IoT network performance, including throughput, energy consumption, latency, packet delivery ratio, and network longevity. We achieve these improvements using techniques such as device authentication, energy-efficient clustering, environmental monitoring using Circular-based Hidden Markov Model (C-HMM), data verification using Awad’s Entropy-based Ten-Fold Cross Entropy Verification (TCEV), and data confidentiality using Twine-LiteNet-based encryption. We employ the Search and Rescue Optimization algorithm (SRO) for optimal route selection, and the encrypted data are securely stored in a cloud server. With extensive network simulations using ns-3, our approach demonstrates substantial enhancements in the specified performance metrics compared with previous works. Specifically, we observe a 20% increase in throughput, a 15% reduction in packet drop rate (PDR), a 35% improvement in network lifetime, and a 10% decrease in energy consumption and delay. These findings underscore the efficacy of our approach in enhancing IoT network interoperability and protection, fostering improved patient care and diagnostic capabilities.

## 1. Introduction

Interoperability is the biggest challenge in the Internet of Things (IoT). Currently, it is one of the main issues in the interconnected Internet of Industrial Things (IIoT), since industrial devices are enabled to provide seamless communication, among others. Therefore, interoperability is required [1,2]. The number of connected devices was projected to reach 20.4 billion by the year 2020, and it has been anticipated to grow to 75 billion by 2025. These interconnected devices require interoperability, security, and seamless and controlled data exchange among devices, and this is referred to as interoperability [3,4]. The energy constraint is a key issue in IIoT devices. Currently, various cluster-based networks are considered to reduce the usage of energy [5]. Still, the energy constraint is a significant issue in the IIoT [6]. Another issue is that most of the cluster-based works are executed in homogeneous environments and residual energy or distance parameters are used for cluster formation [7]. To overcome this issue, heterogeneous sensor-based clustering has been a focus of research [8]. In addition, intelligent routing is a noteworthy phenomenon to improve the quality of service (QoS) during interoperable data communication [9]. Managing the QoS to improve network performance and scalability as well as security solutions was recently addressed using blockchain technology [10]. Blockchain is a decentralized technology that addresses single-point failures [11,12]. However, resources are required to communicate with other IIoT devices. In general, insufficient resources cause huge packet loss or drop [13,14]. Artificial intelligence (AI) plays a vital role in recent IIoT networks, where data of large size are generated and collected from a variety of devices [15]. They are widely used in different smart applications, such as smart cities, healthcare, industry, and so on [16]. In this study, AI acts as an intelligent agent to learn the IIoT environment and collect data from real-world scenarios [17]. AI requires training to classify data from multiple devices, but it often does not ensure reality [18]. Due to the environmental conditions, data collected with IIoT sensors require training for accurate classification. Support Vector Machine (SVM) is one such AI algorithm created for data classification [19].

Data mining techniques for patient health analysis have focused on the application of artificial intelligence techniques for analyzing healthcare data during the COVID-19 pandemic [20]. The effectiveness of deep learning models in analyzing such data is proved and potentially offers valuable insights for diagnostics and treatment [21]. The privacy-preserving techniques used in disease prediction systems utilize deep learning, and they explore various approaches, such as cryptographic techniques, attribute-based encryption, homomorphic encryption, and hybrid methods [22].

- To achieve interoperability for heterogeneous IIoT environments, reliable network connectivity is essential.
- To attain the best data collection from IIoT devices, the environment must be known with precision, and the data must be captured with suitable granularity. In this case, fault data are identified.
- To efficiently find the adaptive threshold, the sensed data should be analyzed in real time.
- To improve the network scalability for incorporating a large number of nodes, energy-efficient clusters should be formed.

### 1.1. Contributions

The major contributions of this study are reported below.

The proposed research contributes to the field of the Internet of Medical Things (IoMT) by presenting a privacy-enhanced illness diagnostic process for healthcare applications, addressing the challenges of privacy protection and inference attacks. The work also demonstrates improved performance metrics, such as throughput, packet delivery ratio, network longevity, energy-consumption reduction, and decreased latency, compared with the previous approaches, making it a valuable contribution to the field. 

### 1.2. Organization

Section 2 of this paper provides a brief overview of the relevant literature and discusses the shortcomings of previous works. Section 3 describes the proposed interoperable AI-IIoT process flowchart, pseudocode, and mathematical expression. In Section 4, the experimental outcomes and performance of the suggested approach are provided. It is shown that the present approach outperforms the baseline models. The conclusion is made in Section 5.

## 2. Related Works

In this section, three kinds of related works, i.e., energy-efficient network models, and network interoperability modeling using blockchain and without blockchain technology, respectively, are reviewed.

### 2.1. Energy-Efficient Network Models

Heterogeneous sensor networks organized into clusters employ a trustworthy energy-aware routing protocol [23]. The primary objective of this article is to reduce routing costs and increase network lifespan. The parameters of residual power, weight value for round-trip time (RTT), and hop count are used in the proposed routing strategy. However, reliable packet routing depends on CH election, and it requires a lot of power. To improve the network quality of service, the authors of [24] suggested an intelligent routing scheme. Limitations: Nearly 256 ($4^{4}$) fuzzy rules are generated for the network, which requires very large energy. CNN is used to train the network according to conditions such as bandwidth availability, congestion status, and traffic level. Initially, K-means clustering is used to partition the nodes into clusters. The K-value is necessary for this purpose. To perform CH election in a heterogeneous WSN, genetic algorithm-based optimal clustering (GAOC) was presented [25]. The choice of the CH depends on many factors, including the total amount of energy remaining, its proximity to the sink nodes, and node density. Multiple information sinks are placed in the network to decrease the distance of communication between the nodes and the sink node, thereby mitigating the hot-spot issue. GA has been considered a powerful approach to CH election, whereas it does not guarantee the attainment of an optimum solution, just like other meta-heuristics. In [26], the matrix-filling theory was presented for data collection in an energy-efficient way. The main goal of this paper is to reduce latency, and the theory proposed is considered to meet this objective. It also uses cluster formation, and time slots are assigned to each cluster for information transfer; further, it is computed using the matrix-filling theory. However, the matrix-filling theory requires huge energy to fulfill all the operations. The energy coverage ratio clustering protocol (E-CRCP) was designed by the authors in [27] to exploit the regional coverage ratio in a way that decreases the node energy consumption. The CH is selected based on the node’s area coverage, and the optimal number of clusters is determined according to the energy amount of each node. As a whole, this article helps the network to run more efficiently, distribute its load more evenly, and last longer. However, if the distance from the node is very high, high energy is needed. In such cases, interoperability fails. 

### 2.2. Security Using Blockchain

IIoT-sensed data are trained using a machine learning algorithm called Support Vector Machine (SVM) [28]. Though SVM is typically applied in real-world applications such as disease diagnosis, it does not directly address the security concerns. To preserve the privacy of IIoT data, a secure SVM approach has been employed by utilizing blockchain-based encrypted IIoT data. Encrypted sensed data are securely transmitted to other nodes without the involvement of a trusted authority. However, it should be noted that homomorphic encryption, which can support complex sensed data, requires significant energy resources, and it may not be suitable for this scenario. 

### 2.3. Security without Blockchain

The authors in [29] proposed a compressed data stream that is generated using low-density parity check (LDPC) code. It has an energy limit. The time it takes to transmit information from a node to a collector is substantial. The issue arises while attempting to process an encrypted data stream coming from a centralized node. The research [30] proposes a method for collecting raw data from Internet of Things gadgets, and it protects users’ anonymity. The fog nodes are trusted, and the message transmitted from the participants is found. However, data privacy is not fully given, since the attackers can easily compromise the fog nodes to obtain the participants’ data. In the study [31], three smart algorithms that can self-learn, adapt to their surroundings, and learn in small increments over time are explored in an Internet of Things setting. Ultimately, an unsupervised method based on a dynamic self-organizing map meets all the criteria. Effective lightweight integrated blockchain (ELIB) was proposed in the paper [32] to accommodate the needs of IIoT gadgets and their users. Security is employed to save sensitive information during transmission [33]. For this purpose, a secure group communication scenario in which the logical trees are constructed for each group was designed. Limitations: The most powerful node must be elected to act as the CH because it plays the main role in the group. Hence, high energy consumption is avoided. The one-way hash function is less strong than the other hashing algorithms. The authors in [34] proposed interoperable and flexible IIoT applications (e.g., smart home). This link utilizes a cloud-based infrastructure and a web of objects to function. To accomplish appliance-to-appliance communication, a Raspberry Pi-based gateway is used.

## 3. The Methodology

**Figure 1:**
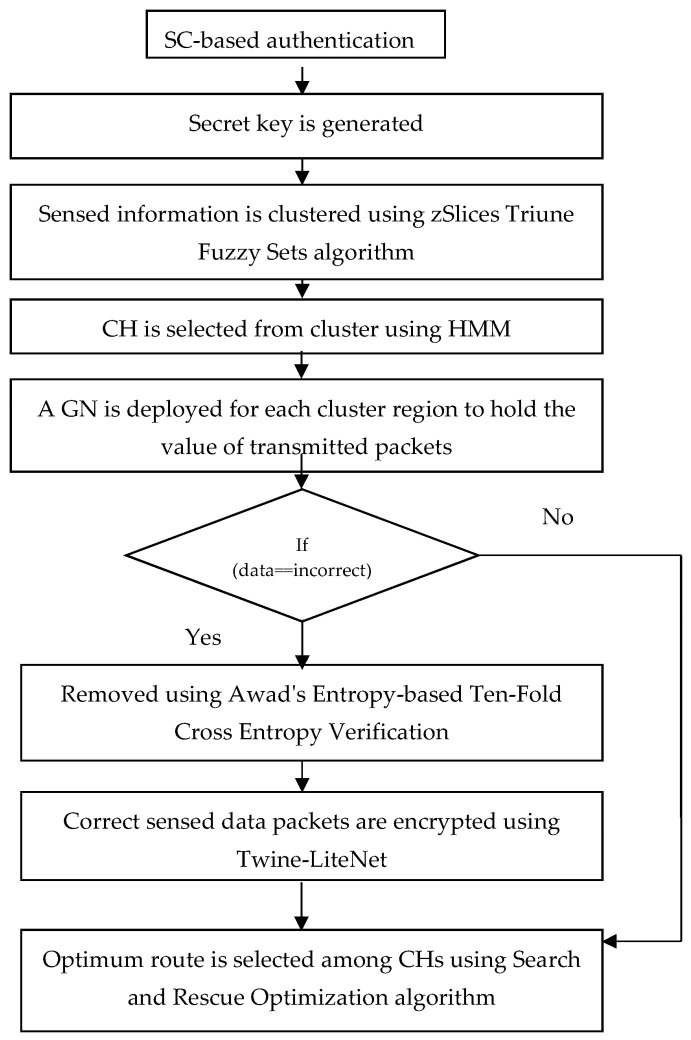
Overall flow chart.

In this research, the problems that exist in the current works are overcome to achieve interoperability. To mitigate the above-mentioned challenges, the proposed model has a three-layered architecture consisting of Perception Layer, Edge Layer, and Cloud Layer, and it comprises IoMT devices (CH and CM) and Guard Nodes (GNs). Figure 1 describes the overall flow chart of the proposed work model. 

### 3.1. Conceptual Model

**Figure 2:**
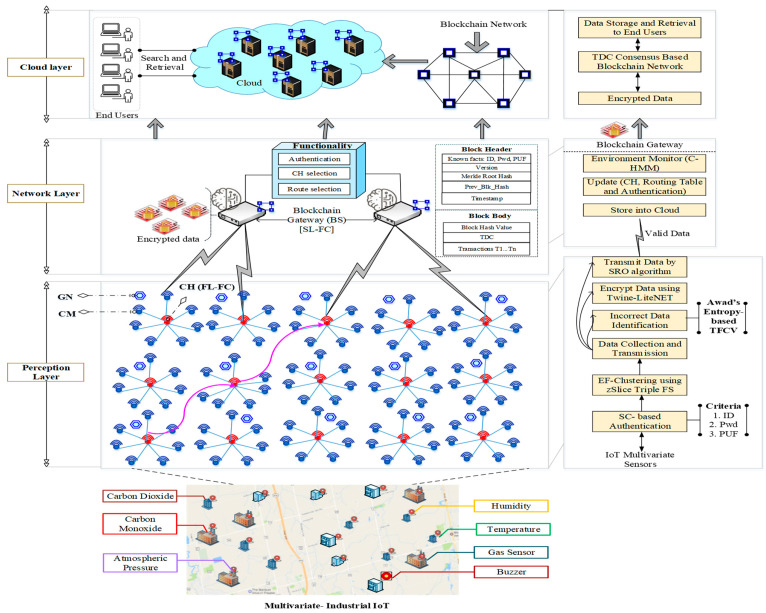
System model.

The architecture of the proposed interoperable AI-IoMT model is presented in Figure 2. The model comprises three layers that explain the overall working of IoMT applications. Figure 1 shows that authentication in the IoT environment is based on Secretkey, and the clustering of sensed data is performed using the zSlices Triune Fuzzy Sets algorithm. Additionally, CH selection is performed using Hidden Markov Model (HMM), and the incorrect data are removed using Awad’s Entropy-based Ten-Fold Cross Entropy Verification, whereas the correctly sensed data packets are encrypted using Twine-LiteNet. Finally, an optimal route is selected among the CHs using the Search and Rescue Optimization algorithm for enabling secure and efficient data transmission in the IoT network.

The proposed method incorporates various security mechanisms to protect patient data and maintain privacy; these are Twine-LiteNet-based encryption and Awad’s Entropy-based Ten-Fold Cross Entropy Verification (TCEV) and may be employed to validate data integrity and reduce the risk of data tampering. 

### 3.2. Secure Credentials (SCs)-Based Authentication

**Figure 3:**
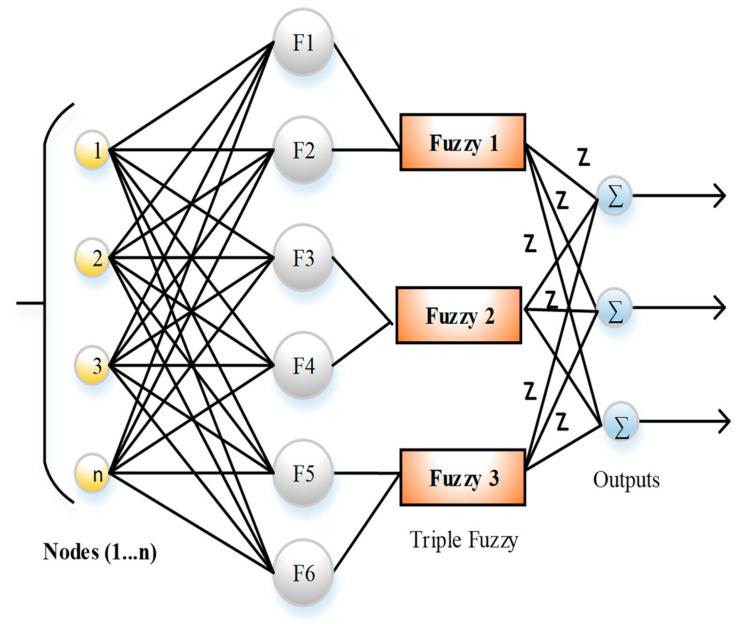
zSlices Fuzzy Sets algorithm.

Initially, each IoMT device is authenticated with its ID, Password, and PUF (physically unclonable function). PUF is a unique identity (digital fingerprint) for each IC. During authentication, these three factors are verified. If they are valid, then the gateway generates a secret key for the device. The secret key is generated using Twine-LiteNet (Lightweight Neural Network). This authentication process is shown in Figure 3. For each authentication operation, these three factors are used as known facts and verified with a secret key for authentication. The proposed TWINE algorithm is executed in the convolutional layer of the Lightweight Neural Network is represented as Algorithm 1.
**Algorithm 1: TWINE**INPUT: ID, password, PUF OUTPUT: Secret key $S_{K}$
$Y_{\left(64\right)}^{1}\leftarrow T_{P}$
${R_{k}}_{\left(32\right)}^{1}\|\ldots .\|{R_{k}}_{\left(32\right)}^{35}\leftarrow R_{k(32\times 36)}$ for i←1 to 35 do $Y_{0(4)}^{i}\left\|Y_{1(4)}^{i}\right\|..\|Y_{14(4)}^{i}\|Y_{15(4)}^{i}\leftarrow Y_{\left(64\right)}^{i}$
${R_{k}}_{0(4)}^{i}\left\|{R_{k}}_{1(4)}^{i}\right\|\ldots \|{R_{k}}_{1(4)}^{i}\leftarrow {R_{k}}_{\left(32\right)}^{i}$ for j←0 to 7 do $Y_{2j+1}^{i}\leftarrow S(Y_{2j}^{i}⨁{R_{k}}_{j}^{i})⨁Y_{2j+1}^{i}$ for k←0 to 15
$Y_{\rho [k]}^{i+1}\leftarrow Y_{k}^{i}$
$Y^{i+1}\leftarrow Y_{0}^{i+1}\left\|Y_{1}^{i+1}\right\|..\|Y_{14}^{i+1}\|Y_{15}^{i+1}$ for j←0 to 7 do $Y_{2j+1}^{36}\leftarrow S(Y_{2j}^{36}⨁{R_{k}}_{j}^{36})⨁Y_{2j+1}^{36}$
$S_{K}\leftarrow Y^{36}$


The TWINE algorithm is a lightweight 64-bit block cipher algorithm. It generates an 80-to-128-bit key that improves hardware efficiency. This algorithm has 16 4-bit sub-blocks. The secure credentials are encrypted using this algorithm, and a secret key $S_{K}$ of 64 bit in length is provided, after collecting plaintext (T_p_). This algorithm of 64 bit in length provides ciphertext (C_T_) of 64 bit in length. It also has a round key (R_k_) value of 80 to 128 bit in length that is derived from S_k_. The TWINE algorithm includes a non-linear layer using a 4-bit diffusion layer and S-Boxes, and it permutes the 16 blocks. The round function is executed 36 times for providing S_k_. The permutation of the block indexes is $\rho :\left\{0,1,\ldots 15\right\}\to \left\{0,1\ldots .15\right\}$, where the sub-block is mapped with the ρ[j]th subblock. We form the clusters by the information sensed from the Environment. In CH election, we consider the six factors: link quality (RSS value) F1, residual energy F2, no. of rounds reached (expected count) F3, fairness score according to geographical area (0-1) F4, coverage ratio F5 and node degree F6.

The pseudocode outlines the TWINE encryption algorithm, which takes inputs such as ID, password, and physically unclonable function (PUF) to generate a secret key (S_K). The algorithm performs a series of operations, including XOR operations, substitution (S), and permutation (ρ), to derive the key from the inputs and the intermediate variables. 

In Figure 3, the Fuzzy Set algorithm includes the *x*-axis and *y*-axis for generating fuzzy rules. In the present study, the third-dimension *z*-axis is taken for fuzzy-set generation; it is known as zSlices and provides the interval set in the third dimension. The representation of zSlices is defined as follows:

(1) $$Z_{i}=\int_{y\in Y}^{i}\int_{v_{i\in J_{iy}}}^{i}\frac{Z_{i}}{y,v_{i}}$$

The membership function of the zSlices Triune Fuzzy Sets algorithm is defined as follows:

(2) $$M_{f}\left(y^{\prime }\right)=\int_{v\in J_{y,}}^{i}\frac{Max\left(Z_{i}\right)}{v},\;J_{y^{\prime }}⫃[0,1]\;$$

where 0≤i≤I, which represents fuzzy set 1. The other two fuzzy-set values are also represented like this. The join operation is performed slice by slice along the *x*-axis. Convex zSlices-based general fuzzy sets P_i_ and Q_i_ are considered with membership grades $M_{fP}\left(y\right)\;and{\;M}_{fQ}\left(y\right)$, and the zSlices-induced fuzzy sets are represented as follows:

(3) $$M_{fP}\left(y\right)=\frac{\sum_{i=0}^{I}\sum_{Pi\in \left[I_{Pi,}s_{Pi}\right]}Z_{i}}{v_{Pi}}$$

(4) $${\;M}_{fQ}\left(y\right)=\frac{\sum_{i=0}^{I}\sum_{v_{Qi^{\prime }\in \left[I_{Qi^{\prime },sQi^{\prime }}\right]}}Z_{i}}{v_{Pi}}$$

**Figure 4:**
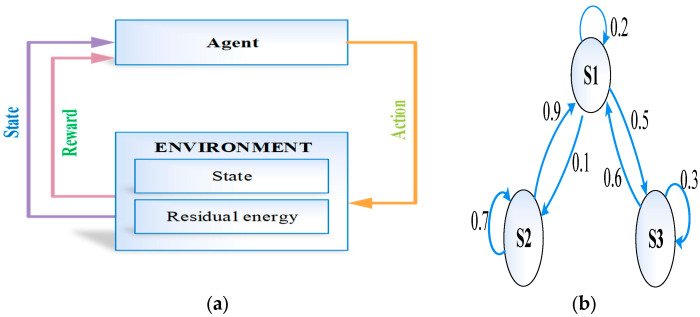

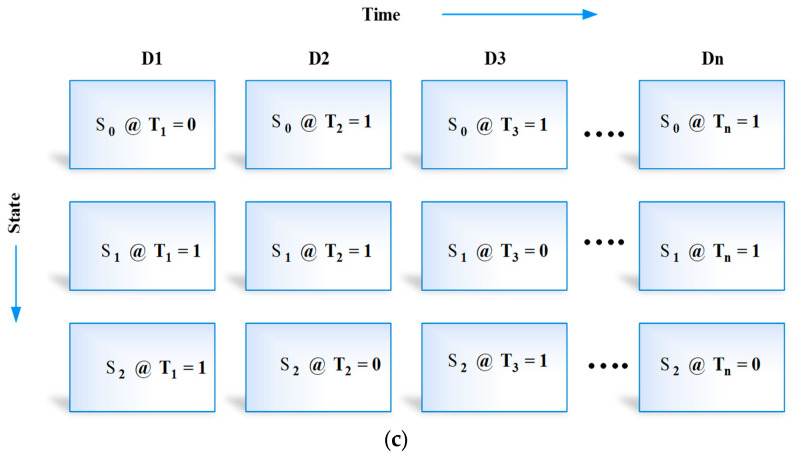
(**a**) HMM, (**b**) time-series state prediction, and (**c**) data readings for sensor nodes.

The join operation between two zSlices-based fuzzy sets is used to reduce the join operation computation between both sets. The other two fuzzy sets are also calculated like this. It is an advanced version of type-2 fuzzy sets. In this approach, triple fuzzy sets are used in parallel mode. Figure 4 shows the diagram for zSlices Triune Fuzzy Sets. Table 1 illustrates the number of fuzzy rules for CH selection. Table 1 also shows the representation of fuzzy rules for cluster head (CH) selection in the proposed research. It outlines the combinations of input variables (F1 to F6) and their corresponding CH selection outcomes (Yes or No), indicating the decision-making process for CH assignment based on the given fuzzy rules.

The threshold for a node $n_{i}$ to become CH is computed as

(5) $$T\left(n_{i}\right)=\left\{\begin{array}{c} \frac{p_{i}}{1\;-\;p_{i}\;\times \;(r\;mod\;1/p_{i})},\;if\;n_{i}\epsilon G\\ 0,\;Otherwise \end{array}\right.$$

In this case, G is the collection of all candidates for CH who have been eliminated in earlier stages. The probability value ($p_{i}$) of each node is computed as follows:

(6) $$p_{i}=p\frac{E_{i}(r)}{\bar{E_{C,l}}(r-1)}$$

where E(r) denotes the residual energy at round r and $\bar{E_{C,l}}$ is the average regional energy of node $n_{i}$ in its cluster C at round r−1. By computing the residual and average energy values, the CH selection probability is computed with the proposed method. The nodes are arranged from the highest to the lowest weight. The median weight value is then used in the following formula to obtain the cutoff value:

(7) $$\mu =\frac{\left(W\left(N_{1}\right)+W\left(N_{2}\right)+\cdots +W(N_{n})\right)}{n}$$

Only the nodes whose weights are greater than a certain threshold (W>) are considered for the next phase. Thus, the number of nodes to be processed in the next stage is reduced based on the weight value. 

After CH selection, a cluster is formed with its cluster members (CMs). All IoMT devices in the region are sensed, and their data are transmitted to the CH. In this step, environmental monitoring is learned, and it is held in the blockchain gateway using Circular-based Hidden Markov Model (C-HMM). The main aim of this algorithm is to determine the hidden state that corresponds to the output and to observe the parameters from the output. C-HMM includes a set of hidden states h={h1,h2,…hn} at time t for any state. The hidden states are determined based on the output O=(O1,O2,…On} with time *t*. In the present work, C-HMM monitors the environment. Generally, C-HMM includes state emission probability and state transition probability. 

State probability is the probability that is obtained from hidden state $h_{k}(t)$ at time t, and it is the transition to hidden state $h_{j}(t+1)$ at time t + 1, which are represented as follows:

(8) $$TP=P\left(h_{j}\left(t+1\right)|h_{k}\left(t\right)\right)={tp}_{jk}$$

State emission probability is a probability that is received from hidden state $h_{j}(t)$ at time t, and it emits observed states $O_{i}(t)$, which are defined as follows:

(9) $$EP=P\left(O_{i}\left(t\right)|h_{j}\left(t\right)\right)=ep_{ij}$$

Finally, C-HMM finds the current state of each IoMT device and updates its information to the gateway. In addition, a GN is deployed for each cluster region, and its main purpose is to hold the value of transmitted packets. Due to the energy-consumption issue of the CH, the GNs are placed, and they do not transmit any information, but they communicate with the CH for avoiding security risks.

With the use of current sensor measurements and environment data, incorrect data are identified and removed from the edge. To find this information, Awad’s Entropy-based Ten-Fold Cross Entropy Verification (TCEV) has been proposed. This method computes the entropy value for each sensed datum. To determine the current sensed data, entropy is computed and compared with the ten sets of the last transmitted data entropy values. This process is held in the CH. 

Then, the correct sensed data packets $\left(D_{p}\right)$ are encrypted using a lightweight cryptography algorithm called Twine-LiteNet (Lightweight Neural Network), which is represented as Algorithm 2. LiteNet is a type of lightweight algorithm that consists of six layers: convolutional layer, LiteModule convolutional layer, dense layer 1, dense layer 2, and softmax layer. To reduce the time consumption of encryption, the aggregated sensed data are encrypted in parallel mode. The convolutional layer of the proposed LiteNet model includes a linear filter that is used to reduce the computational cost of the convolutional layers during encryption. Table 2 describes the shuffle and hexadecimal values of the S-Box. The values are used to encrypt and decrypt the input blocks.
**Algorithm 2: Twine-LiteNet**INPUT: $D_{P}$ OUTPUT: $E_{D}$ Begin { Initialize $D_{P}$ // convolutional layer for i from 1 to n do for j from 1 to n do{ encrypt the data packets $D_{P}$ using TWINE $Y_{64}^{1}\leftarrow D_{P}$ for i←1 to 35 do $Y_{2j+1}^{36}\leftarrow S(Y_{2j}^{36}⨁{R_{k}}_{j}^{36})⨁Y_{2j+1}^{36}$
$E_{D}\leftarrow Y^{36}$ } // Fully connected layer (Lite module, 2 dense layers, and softmax layer) for i from t to n do temp = 0 for j from 1 to n do $temp=temp+w_{ij}\times X[j]$ end for $Y_{i}=temp$ end for end for end for end

The calculation of the proposed convolutional layers is defined as follows:

(10) $$X\left(n\right)=Y(n)\times H(n)$$

(11) $$\sum_{m=0}^{s-1}X\left(m\right)H\left(n-m\right)$$

where X(n) represents the length of the input data packets, H(n) represents the kernel selection, and Y(n) represents the output value. In this layer, the sensed data packets are encrypted. Then, the proposed TWINE algorithm converts plaintext into ciphertext (encrypted data) of 64 bits by performing the round function. It takes 36 rounds to generate ciphertext. The S-box permutation values are defined in the table. The permutation block indexes are defined as ρ:{0,1,…15}, and they are mapped with ρ[j] sub-block. This is also illustrated in the table.

Then, the lite module includes a 1×1 convolutional layer, and the filter size of the current lite module is 1×1, 1×2, 1×3. The main objective of this module is to reduce the computational cost among the convolutional layers. The lite module is also used to reduce the volume efficiency of the parameters. The 1×1 convolutional layer is used to improve the local and cluster feature map representations. LiteNet considers the sense data packets to be input. It includes one lite module, two dense layers, and one softmax layer, which include five units that are defined as follows:

(12) $$\sum_{i}^{5}S_{i}=1$$

where i=1, 2…5 and $S_{i}$ denotes the probability distribution.

(13) $$Y_{i}=\sum_{n}X_{n}w_{ni}$$

where w represents the weight values of the softmax layer and X represents the output of the upper layer. The final calculation of the softmax layer is defined as follows:

(14) $${\;S}^{i}=\frac{\exp\;(Y_{i})}{\sum_{j}^{5}\exp\;(Y_{j})}$$

Finally, the softmax layer provides the encrypted data packets.

**Figure 5:**
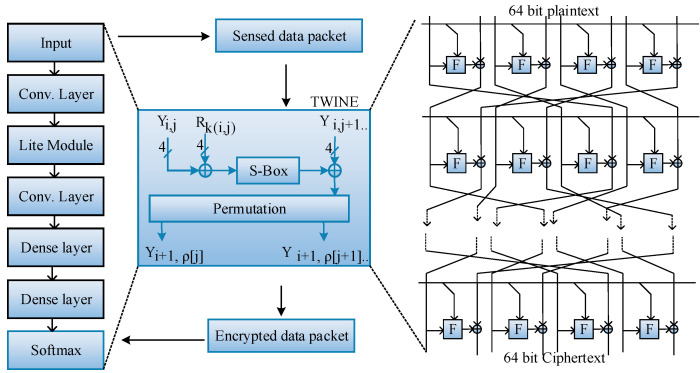
LiteNet with Twine Mode.

The pseudocode describes the encryption process using the Twine-LiteNet algorithm. It encrypts the data packets (D_P) using the TWINE algorithm; then, a fully connected layer (Lite module) is applied to produce the encrypted data (E_D). Figure 5 shows the representation of the flowchart. Later, the optimum route is selected among CHs using four factors: available bandwidth, link quality, residual energy, and path duration. It is selected by the SRO algorithm, and its performance is high when it is compared with other optimization algorithms. 

Single-Objective Optimization (SRO) is chosen for route optimization to focus on optimizing a specific objective, such as minimizing travel distance or reducing delivery time, without considering the conflicting objectives. The motivation behind using SRO algorithms is to simplify the optimization process by reducing the problem to a single objective, by making it easier to find an optimal solution within limited computational time. 

The optimal route is computed by the SRO algorithm. When compared with other optimization algorithms, its performance is high, due to the adoption of clue-based exploration. The clue matrix is formulated, and it consists of route selection attributes and available routes. This can be represented as

(15) $$H=\left[\begin{array}{c} A\\ R \end{array}\right]=\left[\begin{array}{c} \begin{array}{ccc} A_{11} & \cdots & A_{1D}\\ \vdots & \ddots & \vdots \\ A_{N1} & \ldots & A_{ND} \end{array}\\ \begin{array}{ccc} R_{11} & \cdots & R_{1D}\\ \vdots & \ddots & \vdots \\ R_{N1} & \cdots & R_{ND} \end{array} \end{array}\right]$$

where A and R denote the attribute and available routes, respectively. The search direction of the route considering the attributes is expressed as

(16) $${JD}_{x}=\left(A_{x}-R_{y}\right),\;x\neq y$$

where ${JD}_{x}$ denotes the direction of the search for the $x^{th}$ route, and $A_{x}\&\;R_{y}$ denote the $x^{th}$ route position and $y^{th}$ attribute position, respectively. To diversify the change directions to search for a route repeatedly, the binomial crossover mechanism is adopted, and it can be represented as

(17) $$A_{x,k}^{\prime }=\left\{\begin{array}{c} H_{y,k}+v1\times (A_{x,k}-H_{y,k}),\;if\;of\left(H_{y}\right)>of(A_{x})\\ A_{x,k}+v1\times \left(A_{x,k}-H_{y,k}\right),\;if\;k=k_{rand}\;\\ A_{x,k},\;otherwise\; \end{array}\right.\;$$

where $A_{x,k}^{\prime }$ denotes the new position of the $k^{th}$ dimension of the $x^{th}$ route.$\;H_{y,k}$ represents the position of the $k^{th}$ dimension of the $y^{th}$ attribute. $of\left(H_{y}\right)$ and $of(A_{x})$ represent the objective functions for $H_{y}$ and $A_{x}$, respectively. Let v1 denote a random number between 0 and 1. The new position of $x^{th}$ route can be formulated as

(18) $${\;A}_{x}^{\prime }=A_{x}+v2\times \left(H_{y}-H_{r}\right),\;x\neq y\neq r$$

If v2 is a random number from 0 to 1, then the following holds. The boundary conditions are calculated such that the new location is optimal, and they may be expressed as

(19) $$A_{x,k}^{\prime }=\left\{\begin{array}{c} \frac{A_{x,k}\;+\;{A_{k}}^{max}}{2},\;if\;A_{x,k}^{\prime }>{A_{k}}^{max}\\ \frac{A_{x,k}\;+\;{A_{k}}^{min}}{2},\;if\;A_{x,k}^{\prime }>{A_{k}}^{min} \end{array},\;k=1,2,..D\;\right.$$

where ${A_{k}}^{max}$ and ${A_{k}}^{min}$ denote the upper and lower boundaries of the $k^{th}$ dimension.

The above equations are used to search the route in each iteration and the change of the previous position to the new position is stored in the matrix R which can be formulated as,

(20) $$R_{n}=\left\{\begin{array}{l} A_{x},\;if\;of\left(A_{x}^{\prime }\right)>of\left(A_{x}\right)\\ R_{n}\;Otherwise \end{array}\right.$$

The changeover of position is expressed as,

(21) $$A_{x}=\left\{\begin{array}{l} A_{x}^{\prime },\;if\;of\left(A_{x}^{\prime }\right)>of\left(A_{x}\right)\\ A_{x}\;Otherwise \end{array}\right.$$

The pseudo-code for the proposed route selection-based SAR algorithm is presented below as Algorithm 3.
**Algorithm 3: SAR**Population initialization in the range $\left({A_{k}}^{max},{A_{k}}^{min}\right)$ Perform sorting and determine the best solution The routing matrix A takes the first half of the sorted solution and the remaining to matrix R Initialize SE, MF, and FN=0 While the end criterion is not fulfilled do For x=1 to N do Update H using Equation (15) If rand<0.5 do Computation of the position of $x^{th}$ route using Equation (17) Else Computation of the position of $x^{th}$ route using Equation (18) End If Perform boundary conditions of $x^{th}$ route by Equation (19) Updation of matrix R and position of $x^{th}$ route by (20) Updation of FN If FN>MF do $A_{x}$ is replaced with a random solution using Equation (21) End if Perform restart strategy End for Compute the current best position and update the previous best End while Return the best solution 

Finally, the encrypted data are stored in the cloud servers and securely accessed by the end users. Due to the strong edge connection in clustering and routing, interoperability is achieved in information transfer. Similarly, data collection is executed effectively with accurate environment sensing, and it dynamically identifies the fault/incorrect data. Furthermore, blockchain technology is proposed to improve scalability and decentralized communication among IoMT devices. 

The above pseudocode represents the Search and Rescue Optimization (SAR) algorithm for solving a routing problem. It initializes a population, which performs sorting to determine the best solution, and updates the routes based on equations, boundary conditions, and restart strategies until the end criterion is met. The algorithm aims to optimize routing matrix A and achieve the best solution to the given problem.

## 4. Results and Discussion

This section discusses the experimental findings and the suggested interoperable AI-based IoMT. The simulation environment and a case study are the first topics of discussion. Then, the proposed work is contrasted with the current methods. The proposed method enhances security in IIoT environment authentication for device integrity and Twine-LiteNet-based encryption to improve the confidentiality of data packets by providing a global model for illness detection in healthcare applications within the Internet of Medical Things (IoMT) context.

### 4.1. Simulation Study

As part of the modeling of the suggested method, 100 IoMT devices are placed over a 100 m^2^ area and simulated using NS3.26. The machine runs Ubuntu 14.04 and has the NS-3 simulator loaded onto it. Initially, nodes consist of limited energy and are exhausted for each communication. Table 3 portrays the obtained simulation values for the implementation of interoperable network operations, and it represents the simulation parameters and descriptions. 

In this section, a performance analysis is conducted to validate the proposed interoperable IoMT approach with two existing approaches, i.e., EIR-CIoT [35] and BDCS-IoMT [36]. For the proposed interoperable IoMT implementation, two kinds of scenarios are compared, i.e., all-information transfer (with error/fault values) and correct data (without any error readings). In Industry 4.0, fault data event generation and transmission cause a higher number of issues, such as large energy consumption, lower throughput, packet delivery ratio, etc. In the following sub-sections, the evaluation metrics and the simulation results are discussed.

#### 4.1.1. Impact of Throughput 

**Figure 6:**
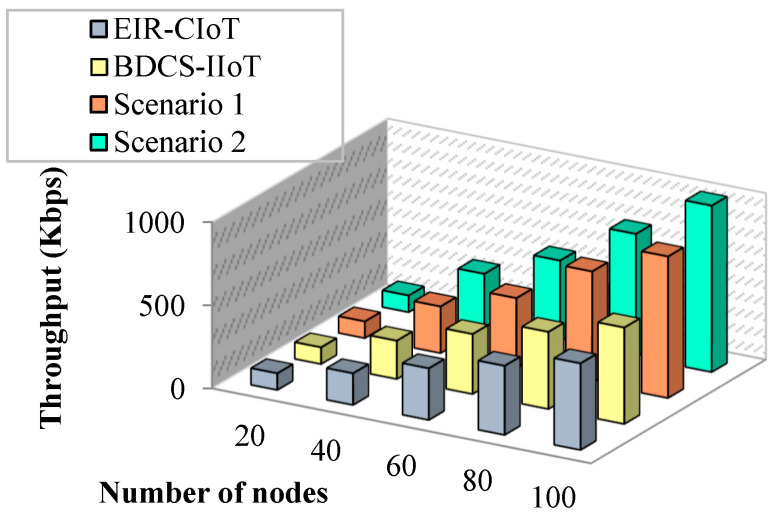
Many nodes vs. throughput.

Throughput is defined as the average number of packets successfully received at the destination node. Figure 6 represents the impact of throughput on the number of nodes. Applying four different deep neural networks for processing the inputs decreases the throughput, and it is implemented in BDCS-IoMT. Likewise, EIR-CIoT uses interoperability and energy-aware routing for throughput optimization. The RSS-based CH is elected for information transfer. RSS with beacon message transmission increases the communication and the computational overhead; hence, the performance of throughput decreases as the number of nodes increases. 

However, uncertainty in data forwarding increases this overhead and reduces the throughput level. This challenge is focused on employing the optimum route using the SRO algorithm. As the objective of routing is lower communication overhead and energy-balanced information transfer, SRO determines the global optimum solution. Further, the elimination of unauthorized nodes in the network and employing authentication decrease the overhead and increase the chance of throughput. For instance, EIRCIoT obtains 190 Kbps throughput for 40 nodes, whereas BDCS-IoMT obtains 230 Kbps throughput for 40 nodes, and 280 Kbps and 320 Kbps for throughput, respectively. 

#### 4.1.2. Impact of Energy Consumption 

The ratio of the total amount of energy used during information transfer is known as the energy-consumption ratio (ECR). The remaining power of a node is calculated using this value. The ECR may be written down as

(22) $$ECR=\frac{AE}{AD}$$

where AE and AD denote the average energy consumption and average information transfer rate, respectively. Further, the number of messages in the buffer is estimated. This parameter is considered if a node has a large number of messages; then, all those data are dropped. This metric is defined as the number of messages that are waiting in the buffer of the node. When the number of simulation runs increases, the energy-consumption rate increases. Minimizing the energy consumption of IoMT devices requires knowledge about the specific traffic in the network and also associated end-to-end communications. Routing packets requires a certain amount of energy to store them in the buffer. Most researchers have found intelligent algorithms for routing packets that improve the network performance in the network layer. In this paper, a study of the energy-consumption usage of wireless devices is presented. The optimization of routing using a simple design in the IoMT environment addresses the energy-consumption problem. Also, IoMT devices and sensors enable not only interoperability among devices but also control in the environment. 

**Figure 7:**
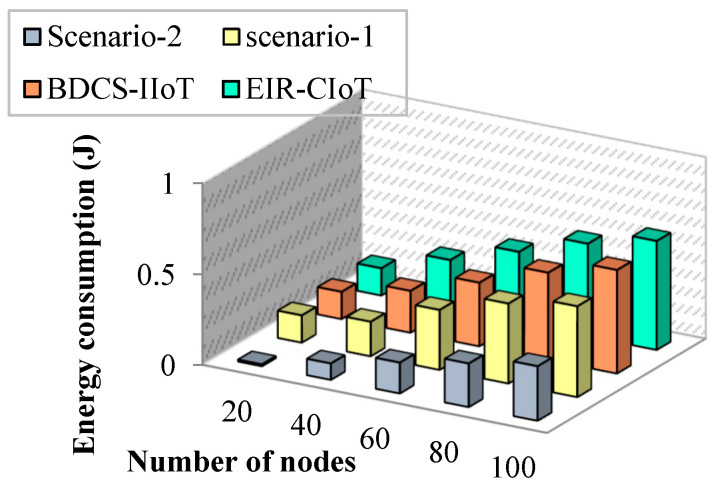
Number of nodes vs. energy consumption.

**Figure 8:**
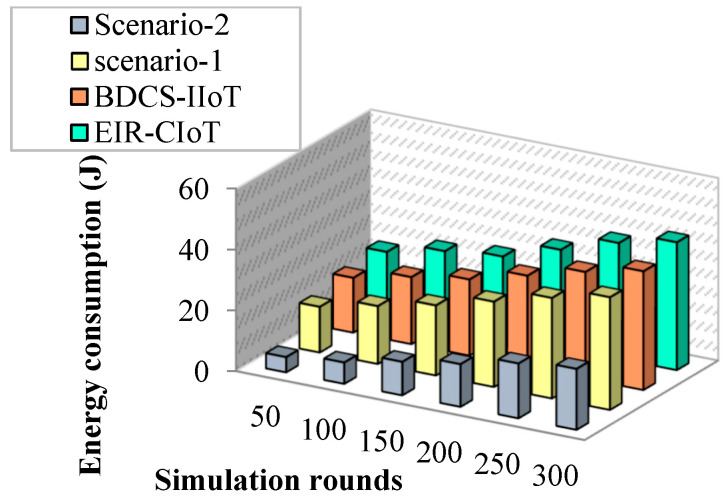
Simulation rounds vs. energy consumption.

The simulation results of energy usage per node are shown in Figure 7. The suggested work reduces power usage by eliminating the faulty data before sending them to the target node, as shown by the simulation results. Further, this is illustrated in Figure 8 based on the simulation rounds. 

#### 4.1.3. Impact of Delay

**Figure 9:**
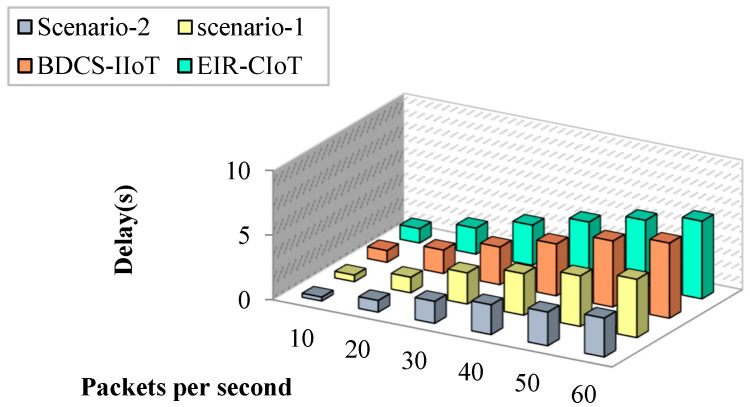
Packets per second vs. delay.

Most energy-saving solutions fail to reduce delay, as has been explored in the literature. Delays in the proposed task are kept to a minimum with the use of optimal CH selection, security provision, and routing mechanisms. Figure 9 shows a comparison of network latency from end to end. According to the findings, the suggested work outperforms prior studies in the field. This study demonstrates that minimizing end-to-end latency cannot be achieved by only cutting energy use. The inefficient information transmission of the EIR-CIoT approach causes a significant delay of up to 5 s. The proposed work has latency of 1 second in an environment of fifty packages per second, and it is much less latency than the EIR-CIoT approach. In addition, the EIR-CIoT approach is narrowly focused on RSS-based routing at the expense of other crucial factors. But with the help of authentication, appropriate CH selection, routing, and packet validation at intermediate nodes, the suggested approach improves the network’s overall performance. As a result, the proposed project achieves a shorter duration of execution than that of similar studies.

#### 4.1.4. Impact of Packet Delivery Ratio 

It is the fraction of a source node’s packets that reach its associated CH node. The PDR, or packet delivery rate, is calculated as

(23) $$PDR=\frac{N_{S}}{N_{R}}$$

**Figure 10:**
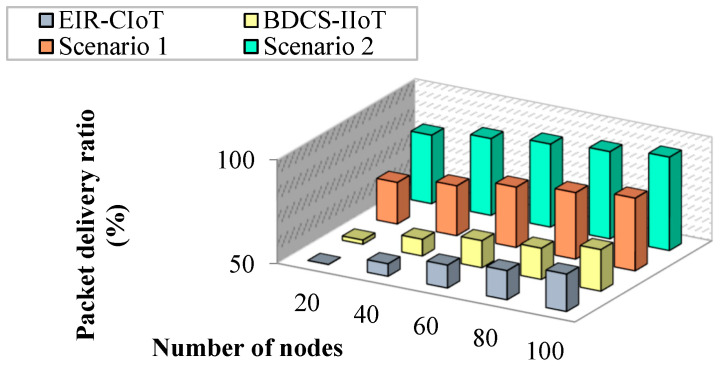
Number of nodes vs. packet delivery ratio.

The percentage of packets effectively delivered to the target node is known as the delivery success ratio. When the data are sent via the best possible route, this proportion increases. Figure 10 shows a comparison between the success rates of the suggested method and those of the existing studies. According to the results, the suggested work has a better delivery success percentage than the existing literature. The proportion of successful deliveries drops as the amount of nodes grows. This is because of the sheer volume of data packets being sent to the network when a big number of nodes is present. As the number of nodes increases, the percentage of successful deliveries decreases across all the works. While the delivery ratio drops by 40% as the number of nodes grows in EIR-CIoT, it drops by only 9% in the proposed work. This study demonstrates that the suggested approach may be scaled to a high number of sites without experiencing any loss of data.

#### 4.1.5. Impact of Network Lifetime 

**Figure 11:**
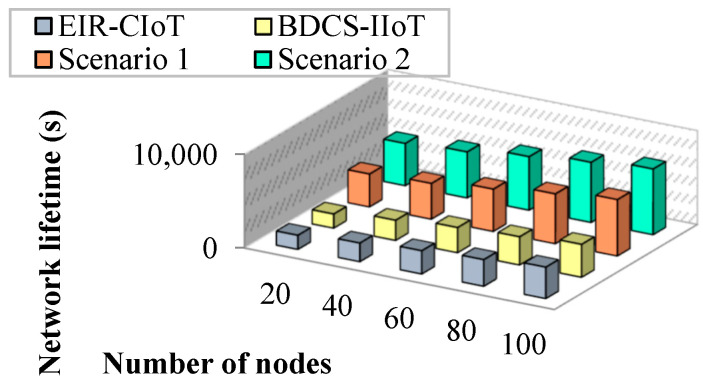
Number of nodes vs. network lifetime.

The effectiveness of the suggested method may be measured, in part, by looking at how long a network lasts. If the network’s energy usage is low, this measure is high.

(24) $$NL=\frac{E_{0}-E[UU]}{P+\delta E\left[Rep\right]}$$

where $E_{0}$ is the starting power used by all sensing nodes; E[UU] is the power that is anticipated to be wasted; and E[Rep] is the power that is expected to be used for reporting, and it is the typical reporting frequency of sensors. The longevity of a network may be quantified in terms of either time or rounds. The comparison of network lifetime is shown in Figure 11. Based on these findings, it seems that the suggested interoperable AI-IoMT strategy extends the lifespan of networks, as their sizes grow. In an area of 50 nodes, the network lifespan with the proposed work is 6000 s, and it is much longer than the previous efforts. As a result, the suggested approach avoids the premature death of nodes and uses less power. While the computational burden does impact the network lifespan, previous techniques such as EIR-CIoT and BDCS-IoMT demonstrate large variations in network lifetime.

The false-positive rate of the proposed method refers to the rate at which the incorrect data are mistakenly identified and removed from the network, and it serves as a measure of its effectiveness in ensuring data accuracy.

A comparison of the effectiveness of the planned and the existing works is shown in Table 4. Throughput, energy consumption, latency, packet delivery ratio, and network longevity are only a few of the metrics that are dominated by the suggested interoperable AI-IoMT solution. The proposed AI-powered method is used for interoperable and secure data collection and routing in the multivariate Industrial IoT. In this process, the obtained throughput is 35% greater than that of EIR-CIoT and 23% greater than that of the BDCS-IoMT approach. The proposed lightweight AI algorithms are suitable for environment-based data collection and transmission. The presented time-dependent consensus (TDC) model can replace PoW and PoS in blockchain technology. 

Table 4 provides a comparison between the suggested method and the existing approaches in terms of performance metrics for fault data prediction and incorrect information transfer scenarios in the Internet of Medical Things (IoMT) context. The suggested method (BDCS-IoMT) offers improved throughput, lower energy consumption, reduced delay, higher packet delivery ratio, and extended network lifetime compared with the existing method (EIR-CIoT). 

**Figure 12:**
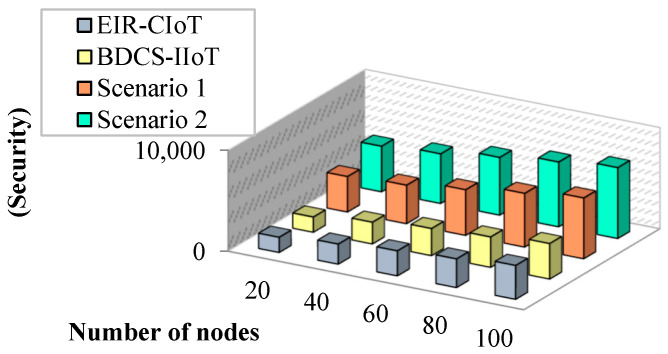
Number of nodes vs. security.

Ensuring the privacy of patient data in an Internet of Medical Things (IoMT) environment is crucial, as flaws in traditional AI systems can expose sensitive information. The comparison of nodes versus security is shown in Figure 12.This research tackles the challenges of energy consumption, latency, throughput, packet delivery ratio, and network longevity in the IoMT setting, and it aims to improve the overall system efficiency and performance.

The developed approach’s performance in terms of compression and computing efficiency could be further improved by including event-driven tools and prospective optimization algorithms [37,38,39]. The integration of these tools could be investigated in the future. 

## 5. Conclusions

Devices in the IoMT environment should possess smooth connectivity and interoperability to achieve maximal efficacy in industrial applications. Moreover, security is also an important aspect to be considered in industrial data. In this paper, both security and interoperability have been achieved in the IoMT environment. Initially, the authentication of IoMT devices is carried out, and the devices are authenticated by the gateway. The clustering of devices is carried out to reduce the energy consumption of the devices, and cluster head selection is performed using the zSlices Triune Fuzzy Sets algorithm based on significant factors. Environmental monitoring is executed to facilitate the effective collection of data by the cluster head (CH), which facilitates interoperability among the devices. The collected data are further examined for correctness using Ten-Fold Cross Entropy Verification (TCEV) in which only the correct data are allowed to transmit to the cloud server. The security of correct data is ensured by implementing Twine-LiteNet, with which the consumption of time for encryption is reduced by operating in parallel mode. The routing of encrypted data to the cloud is performed using the Search and Rescue Optimization algorithm (SRO), and it is performed based on four significant factors. Compared with the counterparts, the suggested technique achieves a 20% improvement in throughput, a 15% reduction in packet drop rate (PDR), and a 35% increase in network lifespan. Furthermore, it achieves around 10% reduction in both the amount of energy used and the amount of latency. In the future, exploring the integration of blockchain technology for enhanced data immutability and privacy, along with investigating the potential of federated learning techniques to further optimize collaborative data analysis and model training, holds promising prospects for advancing the proposed method.

## Figures and Tables

**Table 1:** The Fuzzy Rules.

F1	F2	F3	F4	F5	F6	CH
Low	Low	Low	Low	Low	Low	No
Low	Low	Medium	Medium	High	Medium	Yes
Low	Medium	High	High	Medium	High	Yes
Low	High	Medium	High	Low	High	Yes
Low	Low	Low	Low	Medium	Low	No
Low	Medium	Low	High	High	High	Yes
Medium	Medium	Low	Low	Medium	High	Yes
Medium	Low	Medium	High	High	High	Yes
Medium	Low	Low	Low	Low	Low	No
Medium	High	Medium	Low	Low	Low	No
Medium	High	High	Medium	High	Medium	Yes
Medium	Low	Medium	Low	Low	Medium	No
High	High	High	High	High	High	Yes
High	Low	Low	Low	Low	Low	No
High	High	Medium	Medium	Low	Medium	Yes
High	High	Low	Medium	Medium	High	Yes
High	Low	Medium	Low	High	Low	No
High	Low	Medium	Low	Low	Low	No

**Table 2:** Shuffle and hexadecimal values of S-box.

Shuffle Values of Block			Hexadecimal Values of S-Box	
j	ρ[j]	$\rho^{-1}[j]$	y	S(y)
0	5	1	0	C
1	0	2	1	0
2	1	11	2	F
3	4	6	3	A
4	7	3	4	2
5	12	0	5	B
6	3	9	6	9
7	8	4	7	5
8	13	7	8	8
9	6	10	9	3
10	9	13	A	D
11	2	14	B	7
12	15	5	C	1
13	10	8	D	E
14	11	15	E	6
15	14	12	F	4

**Table 3:** Simulation parameters.

Parameter		Value
Imitation zone		1000×1000 m
Quantity of radar node		100
Deployment		Random
MAC layer		IEEE 802.15.4
Control message		20 bits
Original oomph of node		750 J
Packet amount		400
Retransmission amount		7 (Max)
Size of packet		12 KB
Interval of packet		10 µS
Communication range in sensor		200 m
Rate of data		88 Mbps (Max)
Slots amount		16
Slot period		10 µS
SRO	SE	0.05
	MU	70 D
	R	5
	Number of iterations	100
Number of rounds		100
Simulation time		100 s

**Table 4:** Performance analysis.

Performance		EIR-CIoT	BDCS-IoMT	Scenario-1	Scenario-2
Throughput (Kbps)		307±5.0	346.4±3.0	464±1.0	530±1.0
Energy consumption (J)	Number of nodes	0.384±0.05	0.356±0.03	0.322±0.01	0.162±0.01
	Simulation rounds	30.33±1.5	28.166±1.0	25.833±0.5	12.5±0.5
Delay (s)		3.6±0.5	3.41±0.3	2.61±0.1	1.8±0.01
Packet delivery ratio (%)		59.8±1.5	61.6±1.0	78±0.5	89.4±0.5
Network lifetime (s)		2224±5.0	2561.8±3.0	4620±1.0	5700±1.0

## Data Availability

Data is contained within the article.
